# Glutamic Acid Decarboxylase 65 Antibody-associated Epilepsy and Diplopia: Two Case Reports with Literature Review

**DOI:** 10.1007/s12311-024-01768-w

**Published:** 2024-12-11

**Authors:** Bofei Chen, Yi Shi, Jiahui Guo, Zhiruo Qiu, Beibei Shen, Lina Jiang, Jiajia Fang

**Affiliations:** 1 Department of Neurology, the Fourth Affiliated Hospital of School of Medicine, and International School of Medicine, International Institutes of Medicine, Zhejiang University, Yiwu, 322000 China; 2 Department of Radiology, the Fourth Affiliated Hospital of School of Medicine, and International School of Medicine, International Institutes of Medicine, Zhejiang University, Yiwu, 322000 China

**Keywords:** Epilepsy, GAD65, Diplopia, Cerebellar Ataxias

## Abstract

Glutamic acid decarboxylase 65 (GAD65) antibody-associated epilepsy and diplopia are relatively rare. This article retrospectively analyzed the disease development, diagnosis and treatment process of two cases of GAD65-associated epilepsy with diplopia. Both patients initially exhibited seizures, followed by the onset of diplopia and nystagmus. Due to differences in their diagnostic processes, the two patients showed varying prognoses after treatment. When diplopia and nystagmus are present in patients with epilepsy, these symptoms are often easily attributed to the side effects of antiepileptic medications or not associated with the epilepsy, potentially leading to the oversight of the possibility of GAD65 neurological syndrome. Therefore, clinicians should be aware of the potential association of anti-GAD65 antibodies in epilepsy patients presenting with diplopia, avoidance of missed diagnosis. Furthermore, diplopia and nystagmus may be precursors to ataxia, therefore, when diplopia occurs, proactive treatment should be initiated to prevent disease progression and avoid poor patient outcomes.

Antibodies to glutamic acid decarboxylase (GAD) have been associated with a variety of neurological disorders and type 1 diabetes mellitus, and glutamic acid decarboxylase antibodies were first identified in 1988 in patients with type 1 diabetes mellitus and stiff-person syndrome [1]. Subsequent studies have shown that patients with cerebellar ataxia or drug-resistant temporal lobe epilepsy also have GAD antibodies. GAD is the rate-limiting enzyme for the synthesis of the central nervous system (CNS) inhibitory neurotransmitter gamma-aminobutyric acid (GABA). Anti-GAD65 antibodies can lead to a decrease in the concentration of GABA in the brain, which can lead to a state of hyperexcitability of the CNS, which in turn can lead to a range of clinical manifestations such as limbic encephalitis, epilepsy, stiff-person syndrome, cerebellar ataxia, spinal cord disease and/or brainstem dysfunction [2]. This study details the diagnostic and therapeutic approach employed in two cases of epilepsy associated with anti-GAD65 antibodies, which presented with diplopia.

## Patient 1

A 35-year-old woman presented to our neurology department with refractory epilepsy. There is no family history of epilepsy. At the age of 28, the patient presented with limb convulsions, loss of consciousness, teeth clenching, foaming at the mouth, eye fixation, incontinence, and tongue biting. Each seizure lasted 2–3 min and resolved spontaneously,with gradual improvement in consciousness after the convulsions stopped. The patient was diagnosed with epilepsy and prescribed valproate and carbamazepine for symptomatic management. Since the initial diagnosis, the patient has continued to experience similar symptoms, averaging 5–6 seizures per year, often triggered by mood swings or occurring prior to menstruation. Additionally, the patient reported episodes of panic and anxiety, characterized by brief dreams lasting a few seconds, occurring 3–5 times daily, which could be alleviated.

**Fig. 1:**
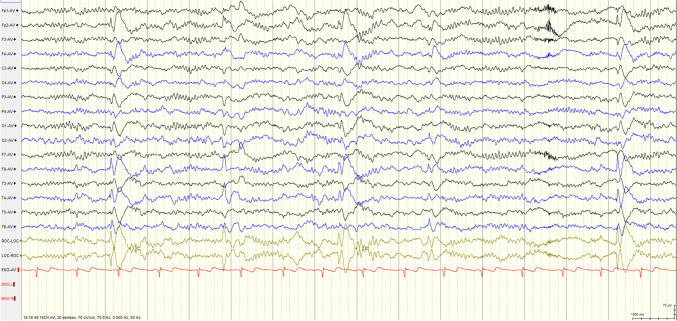
Video EEG showed pathological waves originating from the right frontotemporal region

Complete Blood Count, biochemical tests, antinuclear antibody test, and phospholipid syndrome screening yielded unremarkable results. The serum and cerebrospinal fluid (CSF) IgG levels were measured at 38.9 mg/L. Video EEG analysis revealed moderately abnormal activity during the interictal period, with pathological waves originating from the right frontotemporal region (Fig. 1). Ultrasound examination of the thyroid and cervical lymph nodes identified multiple glial nodules in bilateral thyroid lobes.

An autoimmune brain antibody test yielded positive results, that the patient's serum anti-GAD antibody titer was measured at 1:32, leading to a diagnosis of epilepsy associated with anti-GAD antibodies. Following confirmation of this diagnosis, the patient was administered intravenous immunoglobulin at a dosage of 25 g daily, alongside symptomatic antiepileptic treatment with perampanel.After 4 days of immunoglobulin therapy, the patient developed a rash and leukopenia, leading to the discontinuation of immunoglobulin treatment. The patient was subsequently administered antihistamines and leukocyte-stimulating therapy. During hospitalization, the patient did not experience any seizures, however, abdominal and lower limb rashes persisted.

Two months after discharge from the hospital, the patient developed double vision, particularly noticeable when looking around and to the right side. Monocular vision remained normal, but the right eye exhibited photophobia and a dry sensation. The patient reported tightness and heaviness on the right side of her face and upper limb, along with nausea when looking down and occasional vomiting. Upon visiting local hospitals, MRI and MRA scans did not reveal any significant abnormalities. Considering the potential side effects of perampanel, the patient discontinued the medication independently. However, more than a month later, the symptoms of double vision gradually worsened, and she experienced a sensation of instability while walking. The patient continued to experience seizures during this period and the frequency of seizures did not exhibit a significant change during this time.

Upon admission, the patient exhibited horizontal and vertical nystagmus, with slightly limited abduction of the right eye. There was a reduced pinprick sensation in the right cheek and jaw, while the remainder of the examination was unremarkable.The serum anti-GAD antibody titer increased to 1:100, while the cerebrospinal fluid(CSF) anti-GAD antibody titer was recorded at 1:3.2. Nerve conduction velocity testing, repetitive electrical stimulation tests, cranial MRI, and orbital MR enhancement did not show any significant abnormalities. Dynamic EEG showed a few epileptiform discharges (right middle temporal region).

**Fig. 2:**
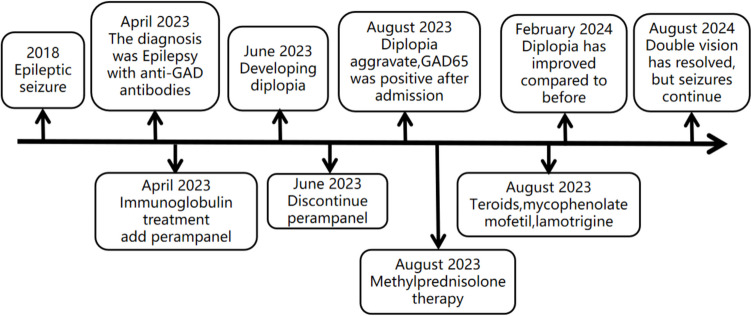
Disease history and therapeutic interventions

Based on the test results, it is assumed that the new symptoms are associated with anti-GAD65 antibodies. The patient was treated with a 3-day course of methylprednisolone therapy, followed by immunomodulation using mycophenolate mofetil. At the time of discharge, the patient still had double vision. Further monitoring and management of her symptoms will be necessary. After discharge from the hospital, steroid therapy was continued, and immunomodulation was initiated with mycophenolate mofetil. Six months later, the patient's diplopia persisted but had improved compared to the initial presentation, while the nystagmus disappeared on examination. By May 2024, after treatment with steroid and mycophenolate mofetil, the patient's serum anti-GAD antibody titer returned to 1:32. Currently, the patient reports no double vision. Despite the initiation of antiepileptic treatment with lamotrigine, the patient continues to experience seizures, however, there has been a reduction in seizure frequency compared to previous levels. Patient history and treatment interventions (Fig. 2).

### Patient 2

A 25-year-old female patient presented with episodic auditory hallucinations and panic attacks that began at age 16. She also experienced loss of consciousness and generalized tonic–clonic seizures, leading to a diagnosis of epilepsy at an external hospital. Initial treatment with lamotrigine and valproate was initiated, but due to inadequate seizure control, the regimen was switched to lamotrigine and levetiracetam.

In September 2022, the patient developed diplopia, with a physical examination revealing limited downward movement of the left eye, and nystagmus.A cranial MRA showed no significant abnormalities, and neuroprotective treatment was initiated; however, the diplopia persisted. The patient exhibited no significant changes in epilepsy symptoms. In December 2023, the patient was hospitalized due to progressive bilateral lower extremity weakness, leading to an inability to stand or walk independently, accompanied by persistent left eye diplopia. Initially, the patient presented with gait instability, characterized by a wide-based gait, which progressively advanced to difficulties in ascending stairs and bilateral lower limb weakness. Examination revealed diplopia during downward gaze, horizontal nystagmus in both eyes, dysdiadochokinesia, a positive Romberg sign, and increased muscle tone in the right limbs, particularly the right lower extremity. A brain MRI showed no notable abnormalities. Dynamic EEG suggested the presence of sporadic sharp waves in the bilateral occipital and posterior temporal regions during sleep. The titers of anti-GAD antibodies in the patient's serum and cerebrospinal fluid are both measured at 1:10, raising suspicion for epilepsy, Stiff Person Syndrome, and cerebellar ataxia. The patient refused immunoglobulin therapy during hospitalization and was treated with clonazepam instead. Post-discharge, the patient's gait instability worsened, prompting treatment with intravenous immunoglobulin in March 2024. As of the current assessment, there was an improvement in both the patient's seizure frequency compared to prior levels, but the patient continues to experience gait instability.

## Review of Literature and Discussion

The first patient presented with epilepsy of unknown etiology and exhibited a poor response to antiepileptic medications. The patient's GAD65 concentration is at a high titer, leading to the diagnosis of anti-GAD65 antibody-associated epilepsy. The patient later developed symptoms of diplopia, and the side effects of perampanel were considered at that time, and the patient stopped the drug on her own, but the symptoms of double vision gradually worsened, and the examination showed horizontal and vertical nystagmus. Re-testing of the brain autoantibody suggested that the anti-GAD antibody titer increased, so diplopia and nystagmus were considered to be related to GAD65. Some literature suggests that diplopia and nystagmus may be the precursor of cerebellar ataxia, so diplopia and nystagmus were considered to be related to cerebellar ataxia [3, 4]. Although the patient did not have obvious manifestations of gait ataxia or limb ataxia, we should consider the patient may have latent autoimmune cerebellar ataxia [5] and be alert to the possibility of symptomatic progression. After immunomodulatory treatment with hormones and mycophenolate mofetil, the patient's diplopia and nystagmus disappeared.

The second patient had a long-standing history of epilepsy and developed diplopia, leading to consultations in both ophthalmology and neurology. Neuroprotective treatment was initiated, resulting in some improvement of the diplopia. One year after the onset of diplopia, the patient experienced gait instability, characterized by a wide-based gait and bilateral lower limb weakness. A comprehensive autoimmune encephalitis antibody panel was conducted, revealing positive GAD65 antibodies. The patient subsequently underwent treatment with intravenous immunoglobulin (IVIG); however, the prognosis was poor, and the patient continued to experience gait instability.

Epilepsy and cerebellar ataxia with anti-GAD antibodies, especially when only manifested as diplopia, is rare. Furthermore, low and high titers of GAD antibodies carry distinct clinical implications. High titers of anti-GAD65 antibodies provide strong support for the diagnosis of anti-GAD-related ataxia and epilepsy, whereas the clinical implications of low titers of anti-GAD antibodies remain ambiguous. Both patients were at a high titer, it may be inferred that epilepsy and cerebellar ataxia are associated with GAD antibodies [5].This article will focus on the clinical manifestations of the disease, treatment, and other aspects in the review, so that clinicians can better understand the disease.

### Etiology and Pathogenesis

Glutamic acid decarboxylase is the rate-limiting enzyme for the synthesis of the central nervous system inhibitory neurotransmitter γ-aminobutyric acid.GAD65 is encoded by the GAD2 gene on chromosome 10, which is expressed predominantly postnatally, and is responsible for the rapid synthesis of GABA required for synaptic transmission [6]. GAD65 is highly expressed in the presynaptic terminals of GABAergic neurons and pancreatic β-cells, mainly in the limbic lobe, brainstem, cerebellum and hippocampus in the brain [2].

The pathogenic mechanism of anti-GAD65 antibodies remains controversial. Anti-GAD65 antibodies are intracellular antigens, and autochthonous immune diseases associated with intracellular neuronal target antigens are mainly associated with T-cell effector mechanisms that can cause neuronal dysfunction and cell death [7]. Previous studies have shown that the number of activated CD8 + T cells and CD4 + T cells in cerebrospinal fluid and peripheral blood is increased in patients with prolonged anti-GAD-associated encephalitis, whereas the number of B cells and plasma cells remains unchanged [8]. However, recent studies have shown that patients with GAD-specific B cells in the CSF have a relatively short disease course, supporting the idea that B-cell or antibody-mediated mechanisms are more pronounced in the early stages of the disease, but even in the early stages of the disease, there are relatively few B cells in the CSF of patients with anti-GAD65 antibody-associated syndrome [9].

### GAD 65 Antibody-associated Epilepsy

GAD65 antibody-associated epilepsy is predominantly seen in female patients, and the age of onset of GAD65 antibody-associated epilepsy is relatively young compared to stiff-person syndrome and ataxia [10]. The clinical manifestations of GAD65 antibody-associated epilepsy can be divided into acute symptomatic epileptic seizures and anti-GAD65 antibody autoimmune-associated epilepsy, which mainly presents as focal seizures [11, 12]. The disorder can present with specific seizure types, and it has been shown that music can induce anti-GAD65 antibody-associated epilepsy [13, 14], which can also manifest as ictal piloerection, and focal epileptic ictal piloerection is a rare symptom that may be overlooked as a subtle sign of a possible association with temporal lobe epilepsy [15]. Patients may present with only ictal piloerection, and in the later stages of the disease, they may present with stiff-person syndrome and generalized tonic–clonic seizures before autoimmune epilepsy is considered [16, 17]. In addition, a patient with GAD65 antibody-associated epilepsy presented with episodic cardiac arrest. It is suggested that patients with rare reflex epilepsy and specific clinical manifestations should be considered for GAD65 antibody testing. It is important to note that the course of anti-GAD65 antibody associated epilepsy is mostly chronic and progressive compared to other autoimmune epilepsies. For this reason, patients with GAD 65 antibody-associated epilepsy may present with isolated epilepsies at the onset of the disease and may not have a combination of psychiatric symptoms such as significant memory impairment and mood disorders, leading to a prolonged course of the disease and may be underdiagnosed, missing the optimal time for treatment [18, 19].

### GAD 65 Antibody-associated Ataxia

GAD 65 antibody-associated ataxia is most common in middle-aged and older female patients, with a median age of onset of 58 years, subacute or chronic onset [20], and is characterised by gait ataxia, followed by limb ataxia, dysarthria and nystagmus [21].

For this reason, patients with GAD 65 antibody-associated epilepsy may present with isolated epilepsies at the onset of the disease and may not have a combination of psychiatric symptoms, and as a result a subset of patients, particularly those with ataxia, develop a range of vestibular and oculomotor abnormalities [22]. Muñiz-Castrillo reported a case of an anti-GAD antibody-positive patient who presented first with acute vertigo and diplopia, then with new-onset gaze-induced nystagmus, and a few months later with downward nystagmus, dyskinesia of the left limb, and gait ataxia. His belief that dizziness and diplopia were transient neurological symptoms before the onset of ataxia [3]. Wang Y's study of anti-GAD antibody-positive patients with vestibular and oculomotor dysfunction found that their early manifestations were mainly dizziness, followed by diplopia. The most common manifestations of vestibular and oculomotor abnormalities were downward gaze and gaze-induced nystagmus [23]. Some studies have shown that about 25% of patients present with episodes of vertigo lasting several months before developing symptoms of cerebellar ataxia and a small percentage of patients will experience diplopia [4]. Therefore, when patients present with transient neurological symptoms of unknown aetiology, such as paroxysmal vertigo and fluctuating diplopia and nystagmus, clinicians should test serum and cerebrospinal fluid for antibodies to GAD, in addition to focusing on the potential for progressive development of ataxia symptoms.

### GAD 65 Antibody-associated Epilepsy and Ataxia

GAD 65 antibody-associated epilepsy combined with ataxia is uncommon in the clinic and the nature of this association remains insufficiently understood. The onset of epilepsy and ataxia may be a simultaneous acute onset or a second symptom several years apart. The majority of patients present with epilepsy followed by ataxia, with the predominant seizure type being generalized tonic–clonic seizures, and all patients present with gait ataxia followed by nystagmus. 50% of patients have type 1 diabetes mellitus and stiff person syndrome. The majority of patients had abnormalities on ancillary tests, including hippocampal sclerosis, temporal lobe hyperintensities, and cerebellar atrophy. After immunotherapy, most patients showed relief from epilepsy and ataxia, with ataxia being more effectively treated. Only one patient died due to tumor progression after hormone therapy. The summary of GAD 65 antibody-associated epilepsy and ataxia is provided in Table 1.

**Table 1:** GAD 65 antibody-associated epilepsy and ataxia

Literature	Sex	Age at first seizure of epilepsy(years)	Age at first seizure of ataxia(years)	Epilepsy onset classification	Clinical manifestations of ataxia	Anti-Seizure Medication	Immunotherapy	Prognosis
Georgieva et al., 2014[24]	M	49	45	GTCS	Cerebellar gait ataxia, dysmetria, nystagmus and mild cerebellar dysarthria	High doses of anticonvulsants	Immunoglobulins, azathioprine, plasmapheresis	Epilepsy and ataxia improved
Maniscalco et al., 2021[25]	M	63	63	GTCS	Ambulation disorder, persistent nystagmus	LEV,LCM	Steroids,intravenous immunoglobulins	Died of a tumor. Ataxia treated better than epilepsy
Dimova et al., 2022[26]	F	37	47	FBTCS, FAS, FIAS	Intermittent gait instability, diplopia, locomotor ataxia, nystagmus, dysarthria	OXC,LEV,VPA,CBZ,LTG,CZP,LCM	Steroids,immunoglobulins, cyclophosphamide, rituximab	ASM and immunotherapy not effective. Surgery improved. Ataxia better with rituximab
Vulliemoz et al., 2007[27]	M	40	58	FIAS, GTCS	Nystagmus, left ataxia and gait ataxia	CBZ,PHT,GBP,VPA,LEV, clobazam	Steroids, azathioprine	Immunotherapy for ataxia is highly effective. Epilepsy improves but still has seizures
Sharma et al., 2016[28]	F	30	30	GTCS	Transient disorientation, diplopia, gait ataxia	LEV, clobazam	Immunoglobulins, azathioprine	Seizure free, ataxia significantly improved
Flores-Cantu et al., 2015[29]	F	15	19	GTCS	Mild tremor, gait and limb ataxia, horizontal lateral gaze nystagmus and dyskinesia	VPA	Steroids, azathioprine	Ataxia and mental status improvement

*M* Male, *F* Female, *FAS* Focal awareness seizures, *FIAS* Focal impaired awareness seizures, *GTCs* Generalized tonic–clonic seizures, *FBTCS* Focal to bilateral tonic–clonic seizures, *LEV* Levetiracetam, *LCM* Lacosamide, *OXC* Oxcarbazepine, *VPA* Sodium valproate, *CBZ* Carbamazepine, *LTG* Lamotrigine, *CZP* Clonazepam, *PHT* Phenytoin, *GBP* Gabapentin

Symptoms of ataxia in anti-GAD65 antibody-associated epilepsy, especially with the onset of diplopia, can easily raise suspicion of ocular disease or antiepileptic drug side effects, leading to ophthalmological consultation or discontinuation of antiepileptic drugs, ultimately resulting in delay in illness or worsening of epileptic symptoms. A case with a history of drug-resistant temporal lobe epilepsy with frequent seizures documented on video EEG and left temporal lobe epileptiform activity. Magnetic resonance showed left hippocampal sclerosis and bilateral mesial temporal lobe hyperintensity. High titres of GAD antibodies in serum and CSF. Steroids, immunoglobulins and cyclophosphamide were ineffective, leading to treatment with selective left amygdaloidectomy, which improved seizures despite memory loss, and five years later the patient developed intermittent diplopia, vertigo and ataxia, which was mistakenly attributed to a new antiepileptic drug, leading to progressive ataxia and transient deterioration, which stabilised with rituximab treatment [26].

Research shows the antibodies associated with epilepsy exhibit distinct epitopes compared to those related to ataxia [30], GAD antibodies derived from individuals with epilepsy demonstrated increased reactivity to the enzyme's C-terminal domain in comparison to those obtained from patients suffering from cerebellar ataxia [31]. A review of the case history indicated the alterations in epileptic symptoms were not significant when the patients exhibited signs of ataxia. Additionally, a comprehensive review of the literature [26, 29–31], in conjunction with our two case studies, it is noteworthy that the age of onset for epilepsy in the majority of cases precedes that of cerebellar ataxia and the two can appear separately. These suggests the seizures and cerebellar ataxia are caused by the different immune mechanism. However, due to the differences in symptom onset, clinicians may find it challenging to correlate these two conditions when patients with epilepsy suddenly present with symptoms such as diplopia and nystagmus.Therefore, when a diagnosis of anti-GAD antibody neurological syndrome has been made and other symptoms are present, it is important to consider the development of other syndromes or autoimmune disorders in addition to the side effects of the medication and to provide timely evaluation and immunotherapy to increase the chances of improvement.The prognostic outcomes observed in our two patients underscore the significance of early intervention in treatment.

### Treatment and Prognosis

Current treatment for anti-GAD65 antibody associated syndromes is mainly symptomatic and immunotherapeutic, and the impact of immunotherapy on the disease and its efficacy is difficult to assess due to a lack of prospective studie [32]. The main treatments for anti-GAD65 antibody epilepsy are antiepileptic drugs, immunotherapy and other treatments such as surgery. Immunotherapy for anti-GAD65 epilepsy is less effective than for other neurological syndromes associated with anti-GAD65 antibodies and other autoimmune encephalitis [33]. However, immunotherapy can reduce the severity and frequency of seizures compared to other treatments, but the effectiveness of treatment decreases with disease duration and side effects, making the treatment of anti-GAD65 antibody-associated epilepsy difficult. Some studies have shown that GAD65 causes neuronal damage mainly by cytotoxic T-cell-mediated antigens, and that anti-GAD65 antibodies are distributed in intracellular epitopes, which are unable to reach intracellular antigens, and therefore many patients develop autoimmune-associated epilepsy and show poor responsiveness to antiepileptic drug therapy [10]. Studies have also shown the potential efficacy of ketogenic diets as an adjunct to direct-response intracerebral neurostimulation therapy in the treatment of medically refractory epilepsy [34, 35]. Anti-GAD65 antibody-associated ataxia responds better to immunotherapy than to epilepsy treatment, with approximately 40–50% of patients showing effective symptomatic improvement after immunotherapy. Most patients receive steroids, and some patients also show symptomatic improvement with immunoglobulin therapy or combined plasma exchange and rituximab therapy [36]. Although much of the available evidence suggests that the disease responds poorly to immunotherapy, early aggressive immunotherapy with symptomatic management such as antiepileptic drugs and late maintenance therapy is still recommended, and long-term follow-up has shown that these measures contribute to remission of clinical symptoms and reduction in relapse. Overall, the disease has a long course and relatively poor immunotherapy outcome, and early diagnosis and timely treatment are more critical to improve prognosis.

## Conclusions

In conclusion, cerebellar ataxia and epilepsy associated with anti-GAD antibodies are rarely observed, particularly in cases presenting with diplopia. Most patients initially exhibit epileptic manifestations, which are subsequently followed by ataxic symptoms, such as nystagmus and gait ataxia. When epilepsy co-occurs with diplopia, clinicians should consider the potential presence of other anti-GAD antibody syndromes, as well as the adverse effects of antiepileptic medications. Additionally, diplopia may serve as an early indicator of impending cerebellar ataxia, and some patients may present with diplopia alone. Therefore, prompt diagnosis and treatment of diplopia with immunotherapy are crucial to prevent disease progression. The management of cerebellar ataxia and epilepsy associated with anti-GAD antibodies is primarily symptomatic and immunotherapeutic; timely immunotherapy is essential for achieving better outcomes.

## Data Availability

No datasets were generated or analysed during the current study.
